# Traditional botanical knowledge of artisanal fishers in southern Brazil

**DOI:** 10.1186/1746-4269-9-54

**Published:** 2013-07-30

**Authors:** Marcela Meneghetti Baptista, Marcelo Alves Ramos, Ulysses Paulino de Albuquerque, Gabriela Coelho-de-Souza, Mara Rejane Ritter

**Affiliations:** 1 Fundação Nacional do Índio, Rua Uruguai, 2648, Bairro Boqueirão, Passo Fundo, Rio Grande do Sul 99.010-112, Brazil; 2 Universidade de Pernambuco, Campus Mata Norte, Departamento de Ciências Biológicas, Rua Prof. Amaro Maltês, 201, Sitio Novo, 55800-000 Nazaré da Mata, Pernambuco, Brazil; 3 Departamento de Biologia, Área de Botânica, Universidade Federal Rural de Pernambuco, Laboratório de Etnobotânica Aplicada, Rua Dom Manuel de Medeiros s.n., Dois Irmãos, Recife, Pernambuco 52171-900, Brazil; 4 Programa de Pós-Graduação em Desenvolvimento Rural, Universidade Federal do Rio Grande do Sul, Av. João Pessoa, 31, Centro, Porto Alegre, Rio Grande do Sul 90040-000, Brazil; 5 Instituto de Biociências, Departamento de Botânica e Programa de Pós-Graduação em Botânica, Universidade Federal do Rio Grande do Sul, Av. Bento Gonçalves, 9500, Bairro Agronomia, Porto Alegre, Rio Grande do Sul 91501-970, Brazil

**Keywords:** Ethnobotany, Plant resources, Artisanal fishers, Riparian community, Rio Grande do Sul

## Abstract

**Background:**

This study characterized the botanical knowledge of artisanal fishers of the Lami community, Porto Alegre, southern Brazil based on answers to the following question: Is the local botanical knowledge of the artisanal fishers of the rural-urban district of Lami still active, even since the district’s insertion into the metropolitan region of Porto Alegre?

**Methods:**

This region, which contains a mosaic of urban and rural areas, hosts the Lami Biological Reserve (LBR) and a community of 13 artisanal fisher families. Semi-structured interviews were conducted with 15 fishers, complemented by participatory observation techniques and free-lists; in these interviews, the species of plants used by the community and their indicated uses were identified.

**Results:**

A total of 111 species belonging to 50 families were identified. No significant differences between the diversities of native and exotic species were found. Seven use categories were reported: medicinal (49%), human food (23.2%), fishing (12.3%), condiments (8%), firewood (5%), mystical purposes (1.45%), and animal food (0.72%). The medicinal species with the highest level of agreement regarding their main uses (AMUs) were *Aloe arborescens* Mill.*, Plectranthus barbatus* Andrews*, Dodonaea viscosa* Jacq.*, Plectranthus ornatus* Codd*, Eugenia uniflora* L., and *Foeniculum vulgare* Mill. For illness and diseases, most plants were used for problems with the digestive system (20 species), followed by the respiratory system (16 species). This community possesses a wide botanical knowledge, especially of medicinal plants, comparable to observations made in other studies with fishing communities in coastal areas of the Atlantic Forest of Brazil.

**Conclusions:**

Ethnobotanical studies in rural-urban areas contribute to preserving local knowledge and provide information that aids in conserving the remaining ecosystems in the region.

## Background

Local knowledge is being lost by many cultures in areas of high biodiversity, especially when activities related to the use of natural resources are gradually abandoned in favor of specializing in market-related activities [1]. Research on the knowledge, use, and management of natural resources by local populations is important because it confirms the value of these cultures and contributes to the self-sufficiency of these populations.

Several researchers have gathered ethnobotanical knowledge from communities located in the coastal area of the Brazilian Atlantic Forest, including the knowledge and use of plants [2-8], the knowledge and use of medicinal plants [9-12], plant management [13], and fishing ecology [1], among others. However, most studies in the coastal area of the Atlantic Forest have been restricted to the southeast coast, and few have addressed fishing communities [14]. Although other studies have examined the ethnobotany of similar groups that practice artisanal fishing (which are characterized by small scale production, the use of small vessels, and work within the immediate family, other relatives or neighborhood groups in which the produce is usually sold to intermediaries without the involvement of industry in marketing and fish processing [15], culturally, these groups can be quite different [14,16].

In Rio Grande do Sul State, southern Brazil, approximately 12,200 active artisanal fishers depend on fishing as their main economic activity [17]. The most extensive lagoon system in Latin America is located on the coastal plain of the state, including the Lagoa dos Patos, also called the “mar de dentro” [“the inland sea”] [18]. Depending on location, fishers living in the lagoon area do not fish in the ocean but share limited fishing territories in the lagoon. The Lagoa dos Patos, according to Kalikoski *et al.*[19], is an important nursery ground for many commercially important species of fish and crustaceans. During the twentieth century, the lagoon was a center of artisanal fishing in southern Brazil and contributed significantly to national fish and shellfish production.

Urbanization is increasingly frequent in developing countries [20]. In these transformations, rural areas begin to be included in the urban area, producing a new category: rural-urban areas.

In Rio Grande do Sul, many fishing communities have experienced this process [21,22]. Simultaneously, several studies have examined artisanal fishing in sea [23,24] and inland [25-28] waters, but none have focused on the ethnobotany of these fishing communities. The present study attempts to answer the following question: is the local botanical knowledge of the artisanal fishers of the rural-urban district of Lami still active, even since the district’s insertion into the metropolitan region of Porto Alegre? In this context, the objectives are as follows: a) to characterize the history of the development of socio-economic activities in Lami, emphasizing artisanal fishery; b) to characterize the local botanical knowledge of the artisanal fishers from Lami and to determine any changes in knowledge in relation to preceding generations; and c) to characterize traditional knowledge about medicinal plants and identifying species that are more important to the fishing communities.

## Methods

### Study area

**Figure 1:**
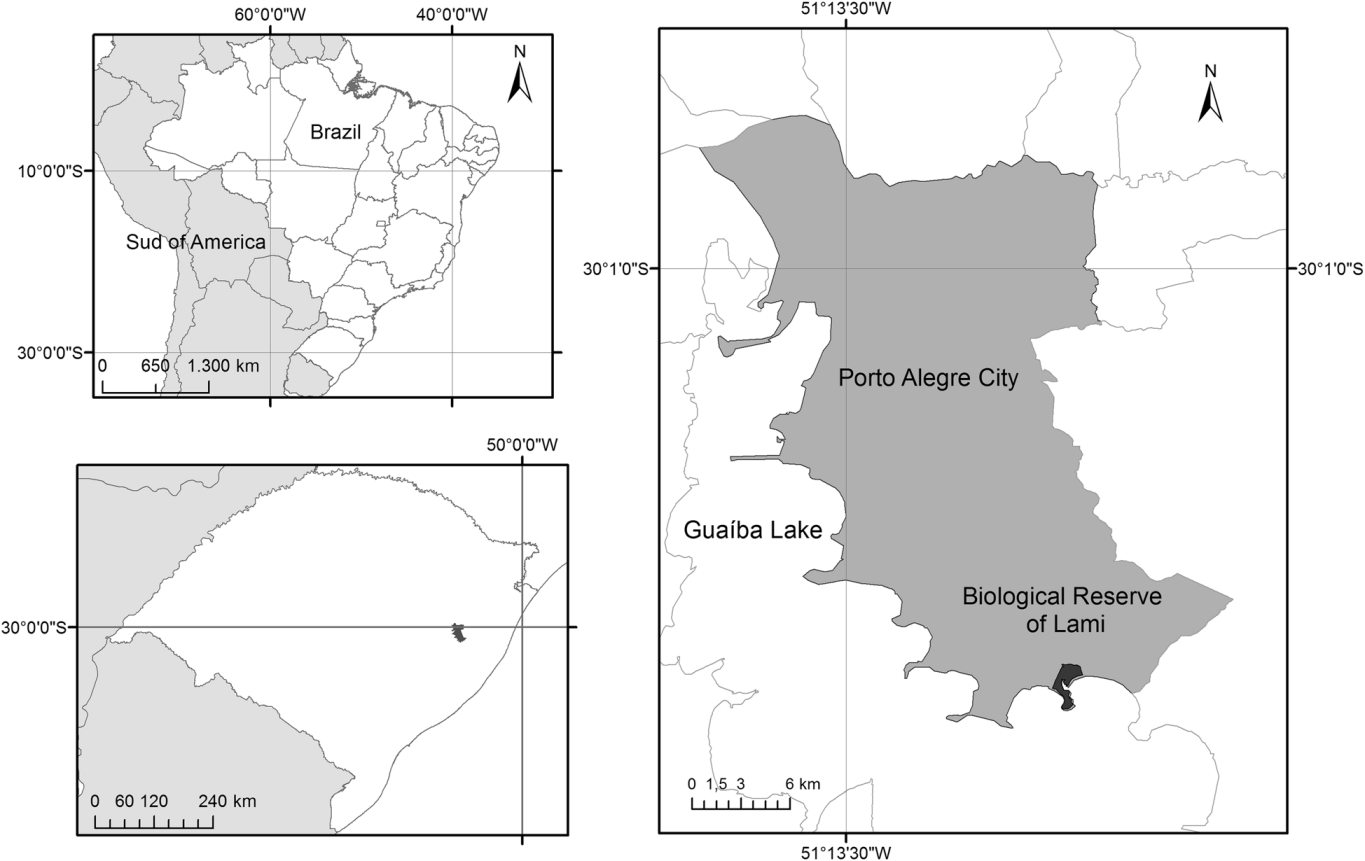
Study area-Lami, Porto Alegre City, southern Brazil.

The study was performed in the neighborhood of Lami, which is situated in the southern part of the city of Porto Alegre (30 km from the city center, with approximate coordinates 30°15’S and 51°05’W) (Figure 1). This neighborhood borders Guaíba Lake and is rural-urban, with small truck farms interspersed between several urban centers [29]. In addition to agriculture, small tradesmen and artisanal fishing persist because an active community of artisanal fishers exist, who are of Azorean descent. This region of the city has undergone rapid urbanization with some accompanying deforestation.

The forest remnants of Porto Alegre are located in this area, and based on the presence of these remnants, combined with the presence of the rare gymnosperm *Ephedra tweediana* Fisch and C.A. Mey, a municipal conservation unit was created: the Lami Biological Reserve (LBR), which protects some unique regional ecosystems and native species of fauna and flora [30]. This part of the municipality of Porto Alegre contains remnants of sandy-soil vegetation, which are characteristic of the pioneer fluvial-lacustrine formation [31].

The implementation of the LBR effectively began in 2000, when it was expanded to 179.78 ha with the preparation of the Participatory Management Plan of LBR; this plan included the participation of the fishing community and the members of the Communitarian Home-based Pharmacy of Lami (CHP) [30]. The CHP was established in 2000 as an initiative to home-produce herbal medicines and was linked to the Movement of Women Peasants of Brazil and hosted in the area of intensive use within the biological reserve. This plan provided a forum to exchange information about regional medical plants, and advice was provided by members of the academic community of the Universidade Federal do Rio Grande do Sul and non-governmental organizations. After seven years, the Lami CHP ceased activity in 2007.

### Data collection

The 13 families of the Lami fishing community were invited to participate in the study during a formal presentation of the project. All families signed a prior consent form (case number: 02000.003729/2008-29 at Conselho de Gestão do Patrimônio Genético/Ministério do Meio Ambiente/Brazil). The history of socioeconomic activity in Lami, particularly artisanal fishing, was studied by surveying literature reviews [6,25,32,33] and conducting semi-structured interviews with fishers.

An ethnobotanical survey was conducted from 2007 to 2008 using participatory observation and semi-structured interviews [34,35]. Information was obtained from a script containing questions about the socioeconomic profile of the informant, the knowledge and use of plant resources in the region, methods of obtaining plant resources (cultivation, extractivism or purchase), and the details of their medicinal uses (the plant part used and the preparation method). Together with the interviews, the free-list technique was also used to assess the richness of known plants that were used by the interview subjects [35].

Fifteen fishers were interviewed (13 men and 2 women, who represented the entire community) about the knowledge and use of plants (in the categories of animal food, human food, condiments, firewood, medicinal, mystical purposes, and fishing), and the interviews were recorded and transcribed.

The plants mentioned were collected during the interviews and processed for deposition in a herbarium following standard ethnobotanical procedures [36,37]. The plants were identified in the Department of Botany, UFRGS, using specialized literature [31,38-41], and the valid plant names were confirmed using The Plant List database [42]. Collected material in good condition was deposited in the ICN herbarium of the Institute of Biosciences/UFRGS. The APG III rating system was used [43].

All the plants were classified according to habit and as native or exotic according to their biogeographical origins. We considered plants that are natural to the region (the state of Rio Grande do Sul, southern Brazil) as native species and those originating from other regions of Brazil, South America, and other continents as exotic species.

The studied species were categorized according to their uses: medicinal, human food, animal food, condiments, firewood, mystical purposes, and fishing. In the last group, we included plants that were used to build boats and temporary fishing camps, to tan cotton fishing nets, and to make buoys (or floats).

The medicinal uses mentioned by the fishers were classified according to disease categories mentioned in the ICD-10-International Statistical Classification of Diseases and Related Health Problems of the World Health Organization [44] (Table 1). In this sense, the uses mentioned by the interview subjects were grouped into 17 categories.

**Table 1:** Categories of diseases mentioned by artisanal fishers of the Lami neighborhood, Porto Alegre, Rio Grande do Sul, Brazil

**Category of disease**	**Mentioned medicinal use**
Abortifact	Abortifact
Aphrodisiac	Aphrodisiac
Mouth and throat	Toothache, gargling, mouth infection
Nutrition and metabolism	Cholesterol, diabetes, weight loss, triglycerides
Skin	Chilblains, wart removal
Circulatory system	Blood thinning, anemia, raised blood pressure, low blood pressure, heart, to unblock veins, palpitations, pressure regulation
Digestive system	Stomach, colic, congestion, diarrhea, abdominal pain, stomach ache, nausea, stomach, liver, gas, laxative, malaise, intestinal problems, antidiarrheal
Genitourinary system	Bladder, cystitis, diuretic, inflammation of kidneys and bladder, urinary tract infection, menopause, increasing lactation, kidney stones, kidney, uterus
Nervous system	Calmative
Musculoskeletal system and conjunctive tissue	Hemorrhoids
Respiratory system	Bronchitis, catarrh, expectorant, influenza, acute influenza, twinge, cold, cough
Parasitic and infectious	Antibiotic, cystitis, fever, infection, inflammation of kidneys and bladder, external infection, internal infection, urinary tract infection, vermifuge
Poisoning	Shingles, against poison, snake bite, insect stings
Skin lesions	Healing, against poison, external infections, skin irritation, bruising (washing), snake bite, insect stings
Neoplasms	Cancer
Hair treatment	Hair, hair loss, itching scalp

### Data analysis

The chi-square test was used to evaluate comparisons between species richness and biogeographical origin, methods of obtaining plants, habits, and categories of use; differences were considered significant at p < 0.05 [45]. For each identified method of obtaining plants and for the habits and categories of use, the existence of differences in the proportions of native and exotic species was tested using a chi-square test. The same test was used to assess differences in the proportions of species and the methods of obtaining them within the usage categories that were identified. The analyses were conducted using BioEstat 5.0 software (Instituto Mamirauá, Brasília, Brazil) [45]. The use categories were analyzed by calculating the agreement of use according to Amorozo and Gély [46], in which the percentage of agreement of the main uses (AMU) was calculated to determine the relative importance of species that were mentioned by the community. The AMU was calculated using the formula: AMU = (IAMU / IAUS) × 100, in which IAMU = the number of interview subjects mentioning the main use and IAUS = the number of interview subjects who mentioned one or more uses for the species.

The AMU value was multiplied by a correction factor (CF) that is used to consider the citation frequency of each species that is related to the most-often mentioned use. Thus, CF = IAUS / number of citations for the most-often mentioned use.

The corrected AMUc was determined using the following equation: AMUc = AMU × CF. Species with an AMUc of greater than 50% have high potential for medicinal use.

## Results

The artisanal fishing community of Lami comprises 15 families, in which the mean age of the subjects was 50 years, ranging from 31 to 75 years. Most subjects had not completed elementary school. Two fishers were natives of Lami, and six were from nearby regions, including other neighborhoods of Porto Alegre and the nearby municipality of Viamão. The remaining fishers were from other cities in Rio Grande do Sul. Most of the subjects had lived in Lami for several years (mean = 35); the oldest resident had lived there for approximately 60 years and had fished for 47 years; the newest resident had fished there for four years.

The fishers mentioned 111 plant species belonging to 50 botanical families, of which Asteraceae and Lamiaceae were the most species-rich (13 and 12, respectively) (Table 2). Both native plants (50 spp.) and exotic plants (61 spp.) were mentioned, with no significant differences between the richness of species in the two categories (χ^2^ = 1.09; p = 0.34).

**Table 2:** Plant species used by the Lami fishing community

**Family species/popular name**	**Habit**	**Origin**	**Source**	**Use**	**Part of plant used**
**Amaranthaceae**					
*Alternanthera brasiliana* (L.) Kuntze/ penicilina, ampicilina	HE	E	CL	ME	LE
**Amaryllidaceae**					
*Allium ampeloprasum* L./alho-poró	HE	E	CL	CO	EP
*Allium fistulosum* L./cebolinha	HE	E	CL	CO	EP
*Allium sativum* L./alho	HE	E	CL	CO	EP
**Anacardiaceae**					
*Schinus terebinthifolia* Raddi/aroeira	AR	N	EX	FI	BA
**Annonaceae**					
*Annona* sp./fruta-do-conde	AR	E	CL	FO	FR
**Apiaceae**					
*Foeniculum vulgare* Mill./funcho	HE	E	CL	ME	LE
*Petroselinum crispum* (Mill.) Fuss. Hoffm./salsa	HE	E	CL	CO/ME	LE/RO
**Aristolochiaceae**					
*Aristolochia triangularis* Cham./					
cipó-milongo ou cipó-mil-homens	CL	N	EX	ME	EP
**Asparagaceae**					
*Aloe arborescens* Mill./babosa	SH	E	CL	ME	LE
*Aloe maculata* All./babosa-folha-gorda	SH	E	CL	ME	LE
*Sansevieria trifasciata* Prain/ espada-de-são-jorge	HE	E	CL	MY	LE
**Asteraceae**					
*Achillea millefolium* L./canforeira	SH	E	CL	ME	EP
*Achyrocline satureioides* (Lam.) DC./marcela	HE	N	PU/EX	ME	FL
*Artemisia absinthium* L./losna	HE	E	CL	ME	LE
*Baccharis* sp./carqueja	HE	N	EX	ME	LE
*Chaptalia nutans* (L.) Pol./cachimbinha	HE	N	CL	ME	LE
*Gamochaeta* sp./transagem	HE	N	CL	ME	LE
*Gochnatia polymorpha* (Less.) Cabrera /cambará	AR	N	PU	FI	ST/AP
*Gymnanthemum amygdalinum* (Delile) Sch.Bip. ex Walp. /figatil	SH	E	CL	ME	LE
*Helianthus annuus* L./girassol	HE	E	PU	ME	SE
*Hypochaeris chillensis* (Kunth) Hieron./radicci, almeirão-do-mato	HE	N	EX	FO	EP
*Lactuca sativa* L./alface	HE	E	CL	FO	LE
*Mikania laevigata* Sch. Bip. ex Baker/ guaco	CL	N	CL	ME	LE
*Tanacetum vulgare* L./ catinga-de-mulata, palma-crespa, palma-de-arnica	HE	E	CL	ME	LE/EP
**Basellaceae**					
*Anredera cordifolia* (Ten.) Steenis/ nó-de-cachorro, palma-gorda, planta-para-anemia	CL	N	CL/EX	FO/ME	LE/ST
**Bignoniaceae**					
*Dolichandra unguis-cati* (L.) L.G.Lohmann /cipó-unha-de-gato	CL	N	EX	ME	EP
*Handroanthus heptaphyllus* (Vell.) Mattos /ipê-roxo	AR	N	PU	FI	ST/AP
**Bromeliaceae**					
*Bromelia antiacantha* Bertol./ bananinha-do-mato	HE	N	EX	FO/ME	FR
**Caricaceae**					
*Carica papaya* L./mamoeiro	SH	E	CL	FO	FR
**C**ombretaceae					
*Terminalia australis* Cambess./ amarilho	AR	N	EX	FI	BA
**Convolvulaceae**					
*Ipomoea batatas* (L.) Poir./ batata-doce	HE	E	CL	FO	RO
**Costaceae**					
*Costus spiralis* (Jack.) Roscoe/cana-do-brejo	SH	E	CL	ME	AP/LE
**Cucurbitaceae**					
*Citrullus lanatus* (Thunb.) Matsum. & Nakai/melancia	HE	E	CL	FO	FR
*Cucumis melo* L./melão	HE	E	CL	FO	FR
*Sechium edule* (Jack.) Sw./chuchu	CL	E	CL	FO/ME	LE/FR
**Equisetaceae**					
*Equisetum hyemale* L./cavalinha	HE	E	CL	ME	EP
**Erythroxylaceae**					
*Erythroxylum argentinum* O. E. Schulz/cocão	AR	N	CL/EX	FW/ME/FI	BA/AP
**Euphorbiaceae**					
*Euphorbia prostrata* Aiton/ quebra-pedra-rasteiro	HE	N	CL	ME	LE
*Euphorbia tirucalli* L./avelã	SH	E	CL	ME	AP
*Manihot esculenta* Crantz/aipim	HE	E	CL	FO	RO
*Sebastiania schottiana* (Müll. Arg.) Müll. Arg./ sarandi	AR	N	EX	FI	AP
*Sebastiania* sp./branquilho	AR	N	EX	FW	AP
**Fabaceae**					
*Apuleia leiocarpa* (Vogel) J.F. Macbr./ grápia	AR	N	PU	FI	ST/AP
*Enterolobium contortisiliquum* (Vell.) Morong./timbaúva	AR	N	PU/EX	FI	ST/AP
*Erythrina crista-galli* L./corticeira	AR	N	EX	FI	ST
*Inga virescens* Benth./angazeiro	AR	N	EX	FI	BA
*Mimosa bimucronata* (DC.) Kuntze/ maricá	SH	N	EX	FW	AP
*Myrocarpus frondosus* Allemão/ cabriúva	AR	N	PU	FI	ST/AP
*Parapiptadenia rigida* (Benth.) Brenan/angico	AR	N	PU	FI	ST/AP
**Geraniaceae**					
*Pelargonium graveolens* L’ Hér./ malva-crespa, malva-cheirosa	SH	E	CL	ME	LE
**Lamiaceae**					
*Cunila microcephala* Benth./poejo	HE	N	CL	ME	LE
*Melissa officinalis* L./menta	HE	E	CL	ME	LE
*Mentha* sp.1/hortelã-branca	HE	E	CL	CO/ME	LE
*Mentha* sp.2/hortelã	HE	E	CL	ME	LE
*Ocimum americanum* L./manjericão	HE	E	CL	CO/ME	LE
*Ocimum carnosum* (Spreng.) Link & Otto ex Benth. /arnica, aniz	HE	E	CL	CO/ME	LE
*Ocimum gratissimum* L./cravo	HE	E	CL	FO/ME	LE
*Origanum majorana* L./manjerona	HE	E	CL	CO/ME	LE/AP
*Origanum vulgare* L./orégano	HE	E	CL	CO	LE
*Plectranthus barbatus* Andrews/boldo	SH	E	CL	ME	LE
*Plectranthus ornatus* Codd/boldo-chileno, boldo-chinês, boldo-cheiroso	SH	E	CL	ME	LE
*Rosmarinus officinalis* L./alecrim	SH	E	CL	CO/ME	LE
*Vitex megapotamica* (Spreng.) Moldenke/trauma	SH	N	EX	FO/ME	BA/LE
**Lauraceae**					
*Cinnamomum verum* J.Presl (Blume)/ canela-de-casca	AR	E	EX	FO	BA
*Laurus nobilis* L./louro	AR	E	CL	CO	LE
*Persea americana* Mill./abacate	AR	E	CL	ME	FR/SE
**Lythraceae**					
*Punica granatum* L./romã	AR	E	EX	FO/ME	FR/HU
**Malpighiaceae**					
*Malpighia emarginata* DC./acerola	AR	E	CL	FO	FR
**Malvaceae**					
*Malva sylvestris* L./malva	HE	E	CL	ME	LE
*Sida rhombifolia* L./guaxumba	HE	N	EX	ME	RO
**Melastomataceae**					
*Leandra australis* (Cham.) Cogn./ pixirica, agulhada	SH	N	EX	FO/ME	LE/FR
**Meliaceae**					
*Cedrela fissilis* Vell./cedro	AR	N	PU	FI	ST/AP
**Moraceae**					
*Ficus cestrifolia* Schott /figueira	AR	N	EX	ME	LE
*Maclura tinctoria* (L.) D. Don ex Steud./tajuva	AR	N	EX	FI	BA
*Morus nigra* L./amoreira	AR	E	CL	FO/ME	LE/FR
**Musaceae**					
*Musa* x *paradisiaca* L./bananeira	SH	E	CL	FO	FR
**Myrtaceae**					
*Eucalyptus* sp./eucalipto	AR	E	PU/EX	FW/FI	ST/AP
*Eugenia uniflora* L./pitangueira	AR	N	CL/EX	FO/FW/ME	LE/FR/AP
*Myrciaria cuspidata* O. Berg/camboim	AR	N	EX	FW	AP
*Plinia peruviana* (Poir.) Govaerts / jabuticabeira	AR	N	CL	FO	FR
*Psidium cattleianum* Afzel. ex Sabine/araçá-roxo	AR	N	CL	FO/ME	LE/FR
*Psidium guajava* L./goiabeira	AR	E	CL	FO/ME	LE/FR
**Oxalidaceae**					
*Averrhoa carambola* L./carambola	AR	E	CL	FO	FR
**Passifloraceae**					
*Passiflora* sp./maracujá	SH	N	PU	FO/ME	FR
**Phyllanthaceae**					
*Phyllanthus tenellus* Roxb./ quebra-pedra-em-ramo	HE	N	CL	ME	LE
**Phytolacaceae**					
*Petiveria alliacea* L./guiné	HE	E	CL	ME	EP
**Pinaceae**					
*Pinus* sp./pinheiro	AR	E	PU	FI	ST/AP
**Piperaceae**					
*Piper* sp./pariparoba	HE	N	CL	ME	LE
**Plantaginaceae**					
*Plantago tomentosa* Lam./ transagem	HE	N	EX	FO/ME	EP
*Plantago* sp./transagem	HE	N	CL	ME	EP
**Poaceae**					
*Avena sativa* L./aveia	HE	E	CL	FO	FR
*Cymbopogon citratus* (DC.) Stapf/ capim-cidró	HE	E	CL/PU	ME	LE
*Saccharum officinarum* L./ cana-de-açúcar	SH	E	CL	AF	ST
*Zea mays* L./milho	SH	E	CL	FO/ME	FL
**Polygonaceae**					
*Persicaria hydropiperoides* (Michx.) Small /erva-de-bicho	HE	N	EX	ME	LE/EP
**Polypodiaceae**					
*Microgramma* sp./cipó-cabeludo	EP	N	CL/EX	ME	EP
**Primulaceae**					
*Myrsine guianensis* (Aubl.) Kuntze/ capororoca	AR	N	EX	FI	ST/BA/AP
**Rubiaceae**					
*Coffea arabica* L./café	AR	E	CL	FO	FR
**Rutaceae**					
*Citrus reticulata* Blanco /bergamoteira	AR	E	CL	FO	FR
*Citrus sinensis* (L.) Osbeck/laranjeira	AR	E	CL/PU	FO/ME	LE/FR
*Citrus* sp./limão	AR	E	CL/PU	ME	LE
*Ruta* sp./arruda	HE	E	CL	ME/MY	LE/EP
**Salicaceae**					
*Casearia sylvestris* Sw./chá-de-bugre	AR	N	CL/EX	ME	BA/LE
**Santalaceae**					
*Jodina rhombifolia* (Hook. & Arn.) Reissek/cancorosa-de-três-pontas	SH	N	PU/EX	ME	LE
**Sapindaceae**					
*Dodonaea viscosa* Jacq./					
vassoura-vermelha	SH	N	EX	FW/ME	LE/AP
**Sapotaceae**					
*Sideroxylon obtusifolium* (Roem. & Schult.) T.D. Penn./coronilho	AR	N	EX	ME	BA
**Solanaceae**					
*Solanum lycopersicum* Lam./tomate	SH	E	CL	FO	FR
*Solanum* sp./infalivina	SH	N	CL/EX	ME	LE
**VERBENACEAE**					
*Aloysia citrodora* Palau/erva-cidreira	SH	E	CL	ME	LE
*Aloysia gratissima* (Gillies & Hook.) Tronc./erva-da-pontada	SH	N	CL	ME	LE

**Habit**: *SH* shrub, *AR* tree, *EP* epiphyte, *HE* herbaceous, *CL* climbing.

**Origin**: *E* exotic, *N* native.

**Source**: *CL* cultivation, *PU* purchase, *EX* extractivism.

**Use**: *AF* animal food, *FO* human food, *CO* condiment, *FW* firewood, *ME* medicinal, *MY* mystical, *FI* fishing.

**Part of plant used**: *ST* stem, *BA* bark/*HU* hull, *FL* floral parts, *LE* leaf, *FR* fruit, *AP* aerial parts, *EP* entire plant, *RO* root, *SE* seed.

**Figure 2:**
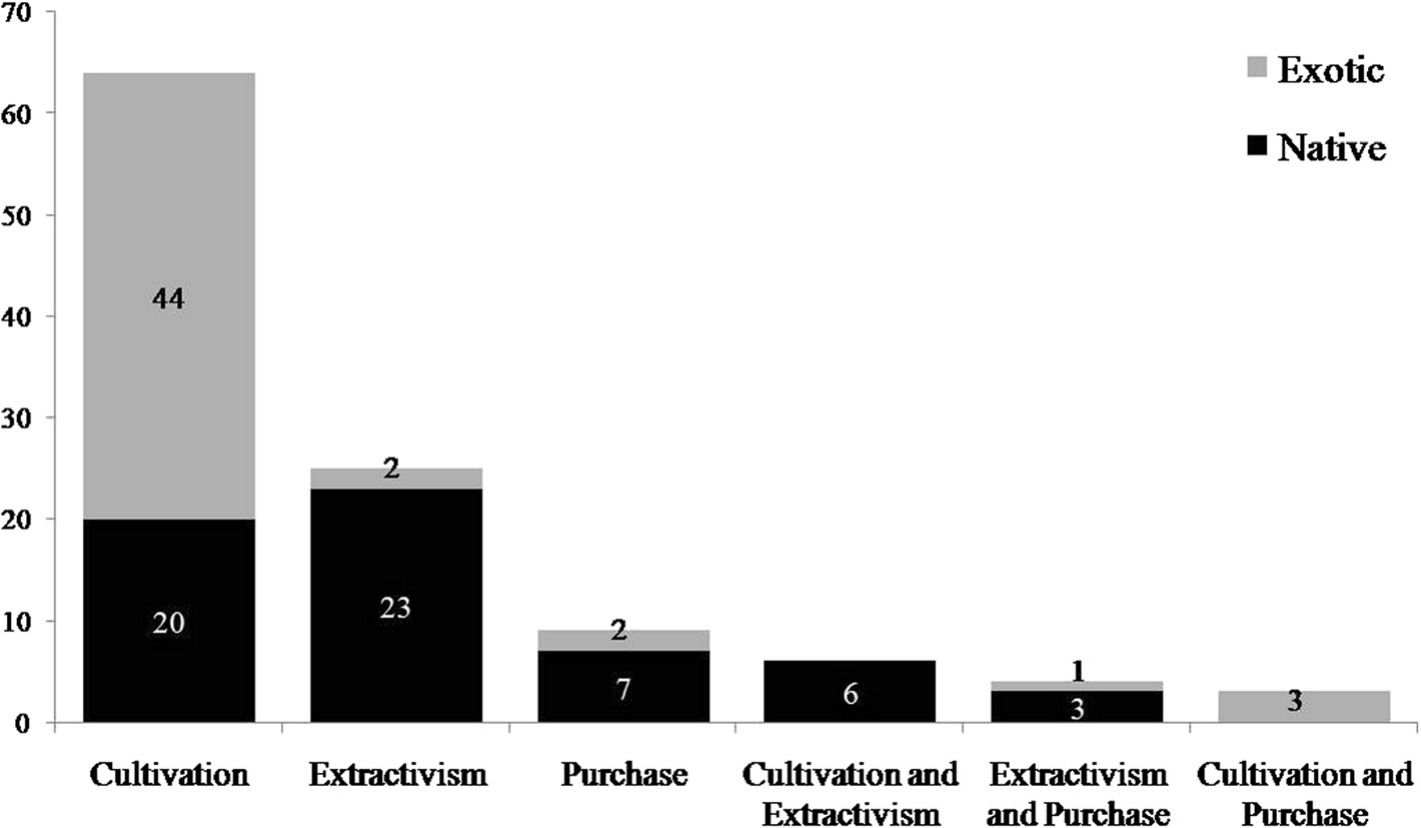
Methods of obtaining plant species mentioned by members of the fishing community of Lami, Porto Alegre, southern Brazil (111 plants mentioned in 15 interviews).

Most species are acquired exclusively by cultivation (64 spp.), extractivism (25 spp.), or purchase (9) (Figure 2). Significant differences were observed in these proportions (χ^2^ = 40.75; p < 0.0001), reinforcing the importance of cultivation as the main method by which the Lami fishers acquire plants. Only 13 species were obtained in more than one way (Figure 2, Table 2).

Most native plants are acquired through extractivism (32 spp.), although a nearly identical number of native plants are cultivated (26 spp.) (Figure 2). These proportions did not differ significantly (χ^2^ = 0.21; p = 0.76), indicating that the use of native plants in the region cannot be understood as a primarily extractivist activity because the arboreous species are obtained by extractivism and the herbaceous species are obtained by cultivation. In contrast, most of the exotic plants are cultivated (47 spp.), and only three are obtained by extractivism in non-managed areas (Figure 2), with significant differences in these values (χ^2^ = 38.72; p < 0.0001). According to our interview subjects, the exotic species collected in non-managed areas include the trees *Cinnamomum verum*, *Eucalyptus* sp., and *Punica granatum* (Table 2).

**Figure 3:**
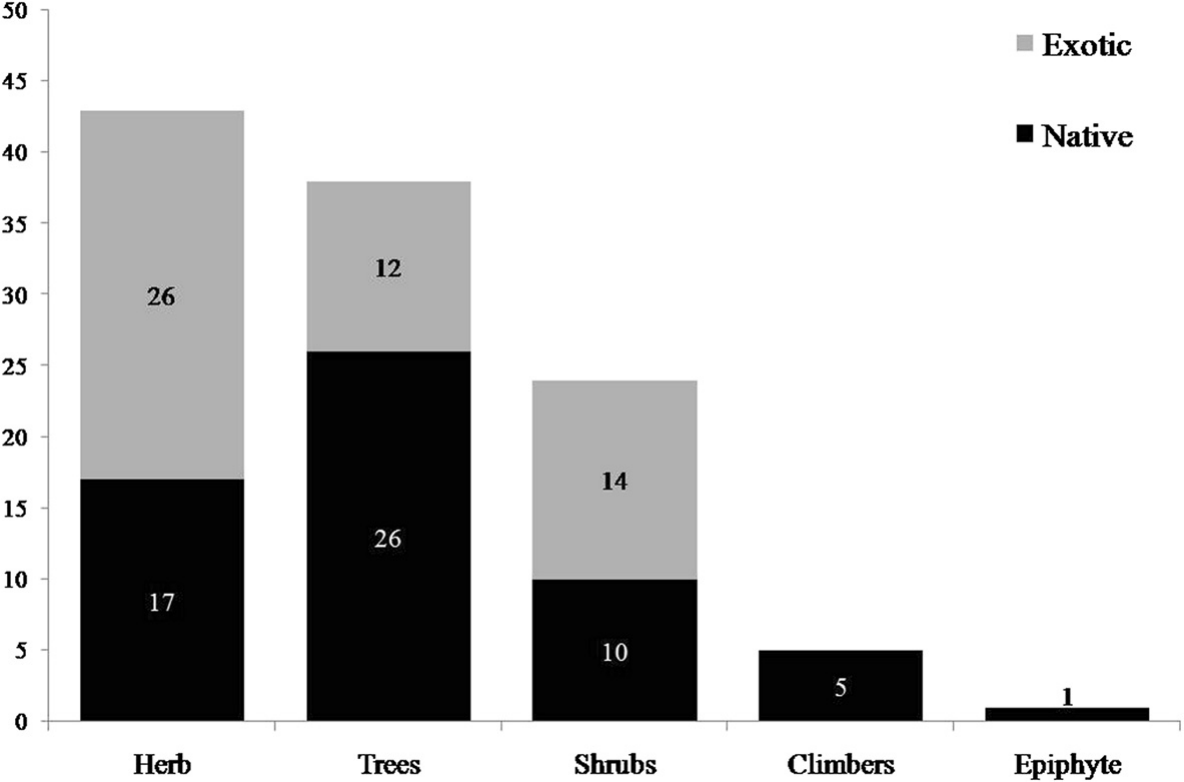
Richness of species grouped by habit that were recorded in the fishing community of Lami, Porto Alegre, southern Brazil (111 plants mentioned in 15 interviews).

Herbaceous plants were the most species-rich group (43 spp.), followed by trees (38 spp.), shrubs (24 spp.), climbers (5 spp.), and epiphytes (1 spp.) (Figure 3). The richness of herbaceous plants was not significantly different from that of trees (χ^2^ = 0,31; p = 0.66) but was statistically greater than that of the remaining groups (p < 0.05). Thus, the proportions of herbaceous and arboreal species mentioned by members of the community are similar. Most of the herbaceous and shrubby plants mentioned are exotic (26 and 14 species, respectively), but this richness is not significantly different from the number of native plants that was recorded for these two groups (17 and 10 species, respectively) (Figure 3) (herbaceous: χ^2^ = 1.88; p = 0.22; shrubby: χ^2^ = 0.67; p = 0.54). Despite this similarity, it appears that exotic herbs and shrubs are most strongly represented in the repertoire of local knowledge. In contrast, the group of arboreal species comprises predominantly native species (26 spp.), significantly more than the richness of exotic arboreal plants (12 spp.) (Figure 3) (χ^2^ = 5.16; p = 0.03).

**Figure 4:**
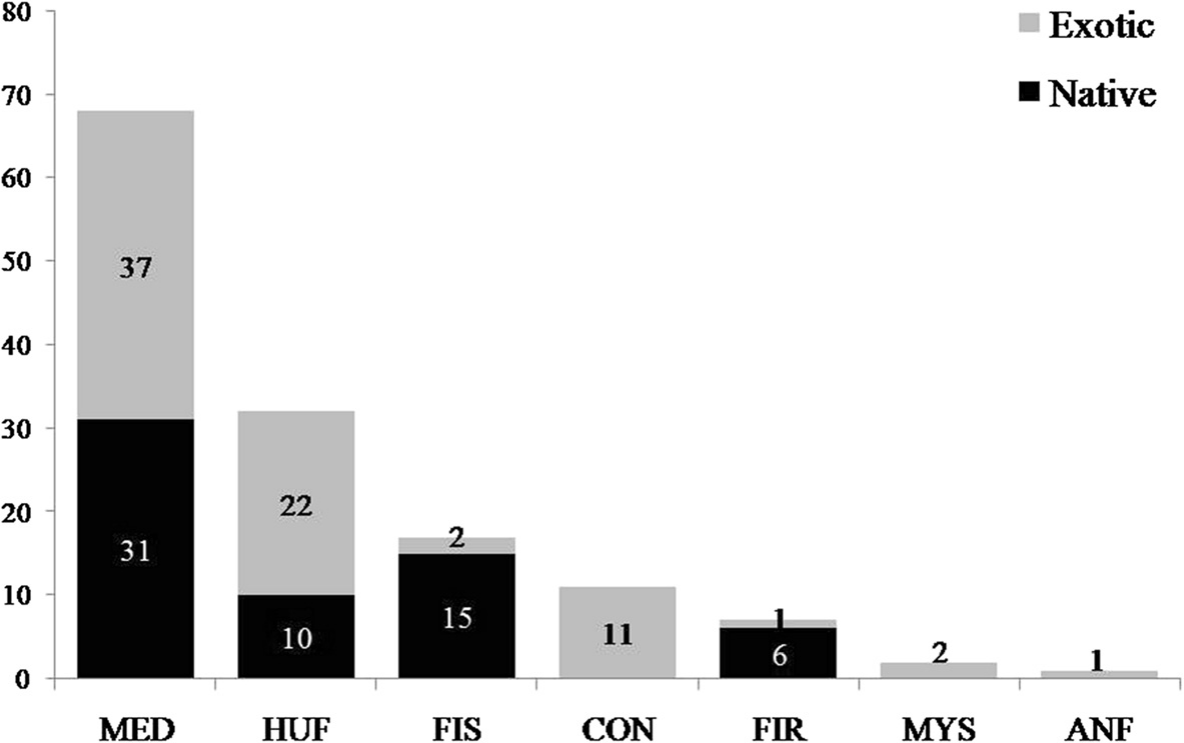
**Usage categories and plant origin mentioned by the fishers of Lami, Porto Alegre, southern Brazil (111 plants mentioned in 15 interviews).** MED = medicinal; HUF = human food; FIS = fishing; CON = condiments; FIR = firewood; MYS = mystical purposes; ANF = animal food.

Most of the plants mentioned by the fishers were collected for medicinal use (69 spp.), followed by use as human food (32 spp.) and use in activities related to fishing (17 spp.) (Figure 4). All 15 interviewed fishers cited plants in the medicinal category. The richness of medicinal species mentioned was higher than that of all other categories identified in this study (p < 0.05). This finding can be understood if we consider that the medicinal category includes a wider variety of indications of use than other categories, requiring a larger repertoire of plants for treatment.

Analysis of the relationship between the origin and the growth habit indicated that among medicinal plants, native and exotic species contribute similar proportions of species (Figure 4) (χ^2^ = 0.36; p = 0.63); this was also true for the human food category. However, species used for fishing activities and as fuel are predominantly native to the region. Plants used as condiments, for mystical purposes, and as animal food are exclusively exotic species (Figure 4).

**Figure 5:**
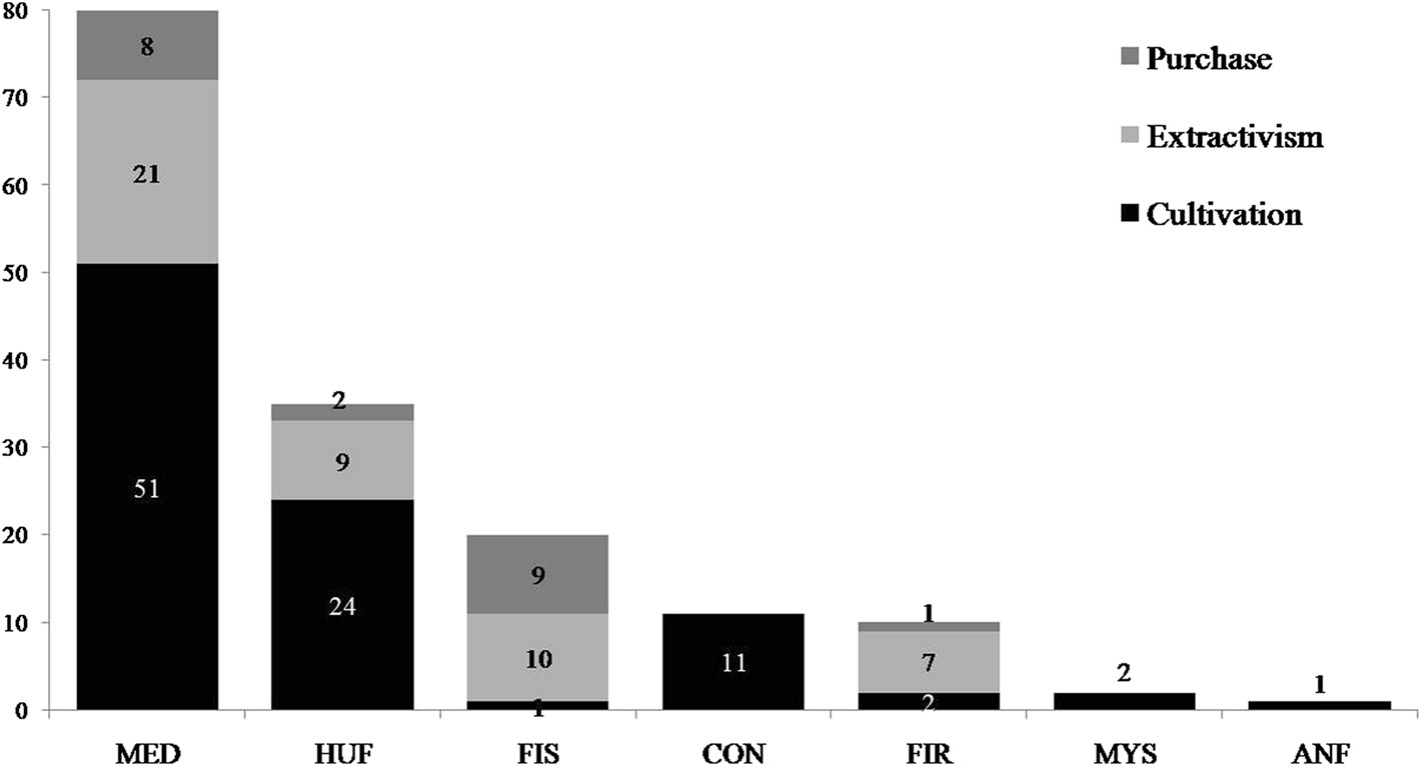
**Usage categories and methods of obtaining plants mentioned by Lami fishers, Porto Alegre, southern Brazil (111 plants mentioned in 15 interviews).** MED = medicinal; HUF = human food; FIS = fishing; CON = condiments; FIR = firewood; MYS = mystical purposes; ANF = animal food.

Comparison between the usage categories and the methods of obtaining the plants showed that the Lami fishers obtain most of their medicinal plants through cultivation (51 spp.), followed by extractivism (21 spp.) and purchase (8 spp.) (Figure 5); these numbers were significantly different (χ^2^ = 36.47; p < 0.0001). Extractivism was the main method used to obtain plants used for fishing and firewood (Figure 5). Few species are used for firewood, i.e., few are subject to this type of harvesting pressure. Curiously, the usage category associated with fishing included the most species acquired by purchase (Figure 5), although these were mostly native species (Figure 4).

### Medicinal use

**Figure 6:**
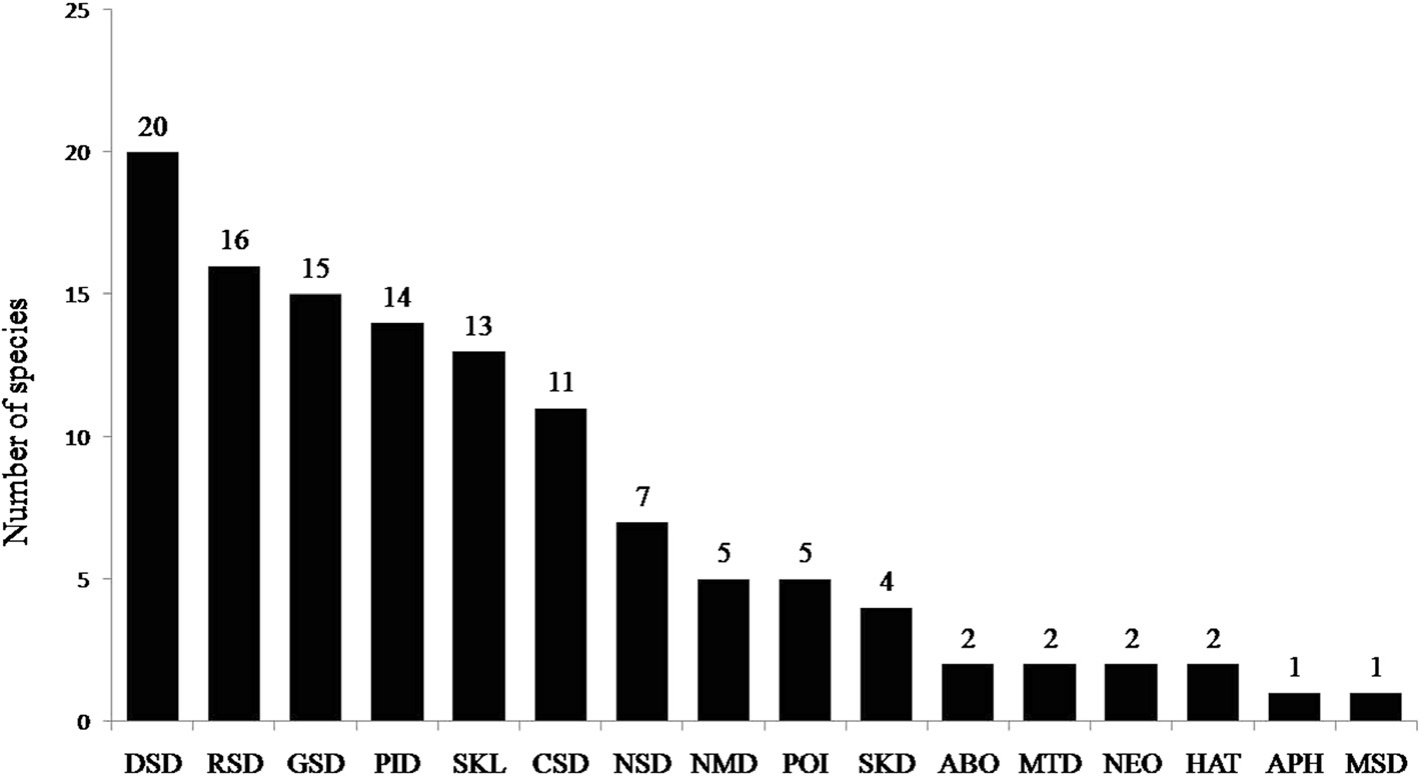
**Distribution of medicinal species by disease category that were mentioned by the fishers of Lami, Porto Alegre, southern Brazil (69 plants mentioned in 15 interviews).** ABO = abortifact; APH = aphrodisiac; MTD = mouth and throat diseases; NMD = nutrition and metabolism diseases; SKD = skin diseases; CSD = circulatory system diseases; DSD = digestive system diseases; GSD = genitourinary system diseases; NSD = nervous system diseases; MSD = musculoskeletal system and conjunctive-tissue diseases; RSD = respiratory system diseases; PID = parasitic and infectious diseases; POI = poisoning; SKL = skin lesions; NEO = neoplasms; HAT = hair treatment.

The largest numbers of medicinal plant species were used to treat ailments of the digestive system (20 spp.), followed by the respiratory system (16 spp.), genitourinary system (15 spp.), parasitic and infectious diseases (14 spp.), skin lesions (13 spp.), and diseases of the circulatory system (11 spp.) (Figure 6).

*Aloe arborescens* was considered the most versatile plant, being indicated for six categories of disease (Table 3), followed by *Foeniculum vulgare, Cymbopogon citratus,* and *Aristolochia triangularis*, each of which was indicated to treat five bodily systems. However, most of the medicinal species mentioned (65%) were indicated for only one ailment category.

**Table 3:** Agreement regarding the main medicinal uses of species

**Species**	**Disease category**	**Number of citations foruse(s) of species**	**Number of citations of the most-mentioned use**	**AMU**	**CF**	**AMUC**
*Aloe arborescens*	Skin lesions, hair treatment, parasitic and infectious diseases, respiratory system, digestive system, neoplasms	21	11	52.4	1.90	100
*Plectranthus barbatus*	Digestive system	8	8	100	0.72	72.72
*Dodonaea viscosa*	Digestive system	7	7	100	0.63	63.63
*Plectranthus ornatus*	Digestive system	7	7	100	0.63	63.63
*Eugenia uniflora*	Digestive system	6	6	100	0.54	54.54
*Foeniculum vulgare*	Digestive system, circulatory system, nervous system, genitourinary system, nutrition and metabolism	11	6	54.5	1	54.54
*Cunila microcephala*	Respiratory system, nervous system, parasitic and infectious diseases	7	5	71.4	0.63	45.45
*Mikania laevigata*	Respiratory system	5	5	100	0.45	45.45
*Achyrocline satureioides*	Digestive system, respiratory system, nervous system	8	5	62.5	0.72	45.45
*Psidium guajava*	Digestive system	4	4	100	0.36	36.36
*Bromelia antiacantha*	Respiratory system	4	4	100	0.36	36.36
*Citrus* sp.	Respiratory system	4	4	100	0.36	36.36
*Anredera cordifolia*	Aphrodisiac, skin lesions, circulatory system	5	4	80	0.45	36.36
*Persicaria hydropiperoides*	Musculoskeletal system and conjunctive tissue, skin lesions, poisoning	5	4	80	0.45	36.36
*Casearia sylvestris*	Circulatory system, nutrition and metabolism, abortive, skin	6	4	66.7	0.54	36.36
*Cymbopogon citratus*	Circulatory system, digestive system, respiratory system, nervous system, parasitic and infectious diseases	12	4	33.3	1.09	36.36
*Aristolochia triangularis*	Skin lesions, poisoning, digestive system, mouth and throat, circulatory system	8	3	37.5	0.72	27.27
*Microgramma* sp.	Genitourinary system	3	3	100	0.27	27.27
*Citrus sinensis*	Respiratory system	3	3	100	0.27	27.27
*Alternanthera brasiliana*	Parasitic and infectious diseases	2	2	100	0.18	18.18
*Psidium cattleianum*	Digestive system	2	2	100	0.18	18.18
*Persea americana*	Hair treatment, skin lesions, skin	4	2	50	0.36	18.18
*Artemisia absinthium*	Digestive system	2	2	100	0.18	18.18
*Sechium edule*	circulatory system	2	2	100	0.18	18.18
*Phyllanthus tenellus*	Genitourinary system	2	2	100	0.18	18.18
*Punica granatum*	Digestive system	2	2	100	0.18	18.18
*Dolichandra unguis-cati*	Genitourinary system, respiratory system, digestive system	4	2	50	0.36	18.18
*Malva sylvestris*	Mouth and throat	2	2	100	0.18	18.18
*Euphorbia tirucalli*	Neoplasms	2	2	100	0.18	18.18
*Aloysia citriodora*	Circulatory system, digestive system, respiratory system, parasitic and infectious diseases	5	2	40	0.45	18.18
*Ruta* sp.	Abortive, poisoning, skin lesions	2	1	50	0.18	9.09
*Petiveria alliacea*	Poisoning, skin lesions	1	1	100	0.09	9.09
*Plantago* sp.	Parasitic and infectious diseases	1	1	100	0.09	9.09
*Plantago tomentosa*	Parasitic and infectious diseases, genitourinary system	1	1	100	0.09	9.09
*Aloysia gratissima*	Respiratory system	1	1	100	0.09	9.09
*Aloe maculata*	Skin lesions	1	1	100	0.09	9.09
*Vitex megapotamica*	Nutrition and metabolism, genitourinary system, circulatory system, respiratory system	4	1	25	0.36	9.09
*Sida rhombifolia*	Genitourinary system, circulatory system	2	1	50	0.18	9.09
*Ocimum carnosum*	Digestive system	1	1	100	0.09	9.09
*Ocimum americanum*	Respiratory system	1	1	100	0.09	9.09
*Gamochaeta* sp.	Skin lesions	1	1	100	0.09	9.09
*Ficus cestrifolia*	Skin	1	1	100	0.09	9.09
*Origanum majorana*	Respiratory system	1	1	100	0.09	9.09
*Rosmarinus officinalis*	Nervous system, circulatory system	2	1	50	0.18	9.09
*Equisetum hyemale*	Circulatory system	1	1	100	0.09	9.09
*Piper* sp.	Genitourinary system, parasitic and infectious diseases	2	1	50	0.18	9.09
*Gymnanthemum amygdalinum*	Digestive system	1	1	100	0.09	9.09
*Euphorbia prostrata*	Genitourinary system	1	1	100	0.09	9.09
*Tanacetum vulgare*	Parasitic and infectious diseases, skin lesions	2	1	50	0.18	9.09
*Pelargonium graveolens*	Parasitic and infectious diseases	1	1	100	0.09	9.09
*Erythroxylum argentinum*	Respiratory system	2	1	50	0.18	9.09
*Zea mays*	Genitourinary system	1	1	100	0.09	9.09
*Petroselinum sativum*	Genitourinary system	1	1	100	0.09	9.09
*Chaptalia nutans*	Skin lesions	1	1	100	0.09	9.09
*Sideroxylon obtusifolium*	Nutrition and metabolism	1	1	100	0.09	9.09
*Costus spiralis*	Genitourinary system, parasitic and infectious diseases	1	1	100	0.09	9.09
*Leandra australis*	Digestive system	1	1	100	0.09	9.09
*Jodina rhombifolia*	Genitourinary system, respiratory system	2	1	50	0.18	9.09
*Baccharis* sp.	Genitourinary system, nutrition and metabolism	1	1	100	0.09	9.09
*Melissa officinalis*	Parasitic and infectious diseases	1	1	100	0.09	9.09
*Mentha* sp.1	Parasitic and infectious diseases	1	1	100	0.09	9.09
*Morus nigra*	Genitourinary system	1	1	100	0.09	9.09
*Solanum* sp.	Digestive system, parasitic and infectious diseases	2	1	50	0.18	9.09
*Mentha* sp.2	Nervous system	1	1	100	0.09	9.09
*Ocimum gratissimum*	Digestive system	1	1	100	0.09	9.09
*Passiflora* sp.	Nervous system	1	1	100	0.09	9.09
*Achillea millefolium*	Skin lesions, poisoning	2	1	50	0.18	9.09
*Helianthus annuus*	Skin lesions, skin	1	1	100	0.09	9.09

Regarding the agreement on the main use (AMUc), *Aloe arborescens* (AMUc = 100%) (used to treat skin lesions), as well as *Plectranthus barbatus* (72.73%)*, Dodonaea viscosa* (63.64%)*, Plectranthus ornatus* (63.64%)*, Eugenia uniflora* (54.54%), and *Foeniculum vulgare* (54.54%) (all used to treat digestive system ailments) (Table 2), exhibited high agreement. These plants may be interesting for pharmacological study regarding their possible therapeutic efficacy. Except for *D. viscosa* and *E. uniflora*, which are native plants acquired by extractivism, all of these plants are exotic cultivated species, emphasizing their importance in the local medical system.

Approximately 56% of the plants that were indicated to have medicinal properties exhibited AMUc values of less than 10% (Table 3). These species were all cited infrequently, i.e., they were known to a few or perhaps only one person. Furthermore, most of these plants are cultivated exotic (52%) or native species that are collected by extractivism (26%).

### Use of plants for fishing

Fishing activities can be performed daily: in the morning, when returning home for lunch, and when checking the material left in the water by late afternoon or early evening. These activities can also be performed for periods of 2 to 15 days, when the fishers camp on the margins of rivers or lakes. During these periods, the fishers usually communicate with an intermediary (produce buyer), who travels every 2 or 3 days to pick up fish. Only half of the people who were interviewed use an engine; the rest fish from rowing boats [17]. The main fishing techniques are the use of sweep nets (circular fishing nets with lead weights on the extremities and a rescue rope in the center) and the use of longlines (many lines with fishing hooks) [47].

The main catches are the “bagre” (*Netuma barba* and *N. planifrons*), “cascudo” (*Hypostomus* spp.), “corvina” (*Micropogonias furnieri*), “grumatã” (*Prochilodus lineatus*), “jundiá” (*Rhamdia* spp.), “linguado” (*Paralichthys* spp.), “peixe-rei” (*Odonthestes* spp.), “piava” (*Lepurinus obtusidens*), “pintado” (*Pimelodus maculatus*), “tainha” (*Mugil* spp.), “traíra” (*Hoplias malabaricus*), and “viola” (*Loricariichthys* spp.) [17].

Four distinct uses of plants were identified for fishing: boat building, making fishing rods, building temporary fishing camps, and making floats for fishing nets. For boat building, the fishers mentioned the “capororoca” (*Myrsine guianensis*), “cambará” (*Gochnatia polymorpha*), “ipê-roxo” (*Handroanthus heptaphyllus*), “grápia” (*Apuleia leiocarpa*), “angico” (*Parapiptadenia rigida*), “pinus” (*Pinus* sp.), “cabriúva” (*Myrocarpus frondosus*), “eucalipto” (*Eucalyptus* sp.), “timbaúva” (*Enterolobium contortisiliquum*), and “cedro” (*Cedrela fissilis*). Some plants that were used as material for boat construction could not be identified because they are purchased by the fishers; these plants were mentioned by the names “canela-preta”, “cedrinho”, and “angelim”. Currently, most of the wood sold in Rio Grande do Sul originates from the north and northeast of the Brazil.

For constructing fishing rods, the fishers mentioned the “sarandi” (*Sebastiania schottiana*), and for building temporary fishing camps, they mentioned the “capororoca” (*Myrsine guianensis*) and “eucalipto” (*Eucalyptus* sp.). According to the fishers, fishing net floats were previously made of “porongo” (*Lagenaria siceraria*) and “corticeira” (*Erythrina crista-galli*), and the weights used were stones or cattle bones. The fishing nets were previously made of cotton, but they are currently constructed from synthetic fibers.

## Discussion

The similarity in the total richness of native and exotic species reveals the dynamism of the local botanical knowledge and an ability to adjust to the requirements of the local people, reflecting a pattern that has previously been documented in the literature. If the Lami fishing community live in an area of strong urban influence, this may contribute to the inclusion of species that are widely used in general society; however, if the fishers live near a biological reserve, this may allow them to have closer contact with native plants. Methods to encourage the use of exotic plants rather than native species have been discussed [48-50], but the data obtained in Lami show that the usage rate of these species is balanced. The same pattern was identified by Voeks [51] while studying the traditional pharmacopeia of an area of the Atlantic Forest in southern Bahia.

In Lami, the plants obtained through cultivation are not restricted to exotic species because many native plants are obtained from fields, backyards, and community gardens. Again, this practice may be related to the presence of the Lami Biological Reserve because although the reserve contributes to the maintenance of local knowledge regarding the native flora, it also limits access to these resources, and the local community must compensate by growing culturally important native species. This response indicates the adaptive nature of traditional botanical knowledge.

The similar richness of useful herbs and trees reveals the balanced proportion of tree and herbaceous species, similar to the balance between native and exotic species. This similarity may reflect a local adaptive process that occurs because the community lives in an area where the use of forest resources is restricted. Thus, it is advantageous to expand the repertoire of knowledge, including species from different habits and biogeographical origins, because this expansion increases the reliability of resources: a temporarily unavailable plant can be replaced by another species. This practice is consistent with the utilitarian redundancy hypothesis, which states that the use of functionally similar species can be part of a strategy to maintain the resilience of local knowledge [48]. Thus, we found that the two most important usage categories to the community (medicinal and food) comprised similar proportions of native and exotic species.

The above discussion characterizes artisanal fishing in the studied region in that published studies have reported that useful flora tend to be dominated by exotic herbaceous species, especially in the medicinal category [49,51-54].

The use of plant species to build boats and construct fishing-net floats appears to have been an important practice in the past in various parts of Brazil. These uses have also been reported by fishers in the state of Alagoas, Northeast Brazil [55]. Traditional plants have been outmoded by new materials, resulting in the loss of this knowledge by fishers in Lami, because few fishers have reported these uses in the past. The same applies to boat building because “timbaúva” (*Enterolobium contortisiliquum*) and “cedro” (*Cedrela fissilis*) were used in Lami, and Hanazaki [14] reported that these species were also used for boat building by “caiçaras,” a term used to describe traditional fishers in southern Brazil.

The predominant medicinal use of plants related to the digestive system, in many cases reflecting the population’s sometimes unhealthy eating habits. Additionally, plants used to treat respiratory-system diseases were commonly mentioned, mainly because of the harsh and variable winter climate in southern Brazil. Similar data were found in other cities in Rio Grande do Sul [56,57] and other parts of Brazil [52,58-60], indicating that the residents use medicinal plants as the first line of treatment for most common diseases.

The plant described as the most versatile, *Aloe arborescens*, was indicated for six disease categories. *A. arborescens* is widely cultivated and used for medicinal purposes in Brazil [40].

According to floral assessments of the region’s biodiversity [31,38,61-66], 594 species have been identified, of which 507 are native (85.35%) and 87 are exotic (14.65%). In this study, the knowledge and use of 113 species by artisanal fishers were recorded, of which 50 are native and 61 are exotic. The preference for exotic species, in principle, indicates that collection does not exert a strong pressure on native species and that use is compatible with conservation. This preference can be explained by the Azorean descent of the fishers. Rio Grande do Sul is largely influenced by European colonization, which implies the use of traditional plants of European origin, mostly for medicinal purposes. Additionally, it is perceived that most species used for medicinal purposes in the fishing community are herbaceous or bushy and more easily collected or cultivated. This pattern of use is found in many communities in Rio Grande do Sul [56,57,67,68].

This paper reveals that the use pattern of plant species by the fishers who live near conservation units in rural-urban areas is compatible with the maintenance of their lifestyles and with conservation, and this use pattern should be encouraged as a conservation strategy for the co-existence of traditional populations in rural-urban areas.

## Conclusions

Although the fishing community of Lami is located in a large city with easy access to modern conveniences, the fishers clearly still value and perpetuate their local botanical knowledge, mainly for medicinal use, human food, and fishing-related uses.

Our interview subjects exhibited good knowledge of the remaining vegetation in the region and of the importance of conservation. They also identified environmental degradation as a problem that affects and reduces their fishing activity.

The rescue of local knowledge and the interest of academic researchers contribute to valuing and respecting the way of life conducted by the traditional fishing community of Lami. In this community, knowledge of the past is combined with knowledge that has been recently incorporated by the community, emphasizing the cultural changes that this area of the city is experiencing.

## Competing interests

The authors declare that they have no competing interests.

## Authors’ contributions

MMB, GCS, and MRR are responsible for the acquisition of study data. All authors have made substantial contributions to the conception and design of the study and to the analysis and interpretation of the data. All authors were involved in drafting and revising the manuscript and approved the final version.
